# All types of recognition errors are (at least partly) attributable to misleading memory evidence, even false alarms

**DOI:** 10.3758/s13423-026-02899-3

**Published:** 2026-03-30

**Authors:** Anne Voormann, Constantin G. Meyer-Grant, Maximilian Luppold, Annelie Rothe-Wulf, Karl Christoph Klauer

**Affiliations:** 1 Department of Psychology, University of Freiburg, Freiburg, Germany; 2 Department of Educational Science, University of Freiburg, Freiburg, Germany; 3 Faculty of Informatics, Heilbronn University of Applied Sciences, Heilbronn, Germany

**Keywords:** Recognition memory, Error-speed effect, Continuous models, Threshold models, Similarity

## Abstract

The error-speed effect, in which items are responded to more accurately if associated with a slow rather than fast erroneous response in a preceding task, is often interpreted as evidence that recognition errors are sometimes driven by systematically misleading memory evidence. However, recent observations challenge this interpretation demonstrating that the error-speed effect only occurs among previously studied items but not among non-studied items (Akan et al. *Memory*, *31*, 1340–1351, 2023), which is not in line with most models of recognition memory. In the present study, we demonstrate that the error-speed effect can occur for non-studied items. In Experiment 1, we replicate the error-speed effect for studied but not non-studied items using picture stimuli. In the second experiment, we systematically manipulate the similarity between studied and non-studied items, thereby increasing misleading memory evidence and the rate of false alarms. As a result, the error-speed effect also emerges among non-studied items, indicating that false recognition decisions for these items are at least partially driven by misleading memory evidence.

## Introduction

The error-speed effect is a well-documented phenomenon in memory research (Starns et al., 2018; Voormann et al., 2021, 2024; Yüvrük et al., 2023), shedding light on the potential origins of recognition errors. It specifies that items associated with fast erroneous responses in an initial recognition task are more likely to be misclassified in a subsequent recognition task compared to items that initially led to slow erroneous responses (Starns et al., 2018). Typically, after a study phase, the first recognition task is a single-item old/new (SION) task, in which participants decide whether a presented item was previously studied (old) or not (new). The second recognition task is most often a two-alternative forced-choice (2AFC) task, in which two items are presented at test – one previously studied and one new – and participants are required to indicate the position of the studied item. But the effect also generalises to other tasks and to different presentation orders (Voormann et al., 2025).

A number of formal recognition-memory models (e.g., the diffusion model and the two-low threshold-model) predict this effect. These models have in common that they assume some kind of systematically misleading memory evidence to account for the error-speed effect. Therefore, the error-speed effect is commonly interpreted as evidence that recognition decisions are at least partially driven by systematically misleading memory evidence, rather than arising solely from random fluctuations in the decision process or incorrect guesses (as assumed by the two-high threshold-model; Voormann et al., 2021).

Recently, Akan et al. (2023) challenged that the effect occurs with both studied *and* non-studied items, as they observed the error-speed effect for old but not for new items. Following the above rationale, this would indicate that misses (incorrect responses to old items) are partially driven by systematically misleading memory evidence, whereas false alarms (incorrect responses to new items) are not. However, formal recognition models that predict the error-speed effect typically predict it for both old and new items.

For example, the diffusion model (Ratcliff, 1978) conceptualises recognition decisions as the result of an evidence accumulation process over time, which proceeds until one of two decision boundaries is reached, triggering the corresponding response. The tendency of the process to approach either boundary is called its *drift rate*. Additionally, response times (RTs) depend on the non-decision time *t*_*0*_, the time for stimulus encoding and response execution, which, however, does not affect the correctness of a response (Voss et al., 2013). According to this model, errors can result from two different mechanisms: they may arise from random fluctuations in the sampling process or from systematically misleading memory evidence (Starns et al., 2018). Errors based on random fluctuation are characterised by a decision process with a drift rate pointing towards the correct response boundary, but due to noise in the sampling process the incorrect boundary is nevertheless reached in some instances. These errors are also called *avoidable* errors because if more evidence had been sampled, the decision process would have crossed the correct response boundary eventually. Errors based on systematic misleading memory evidence, in contrast, are characterised by a drift rate that points towards the incorrect response boundary. These errors are also referred to as *unavoidable* errors because if more evidence had been sampled, the decision process would be even more likely to terminate with an incorrect response. It is these latter errors that cause the error-speed effect for old and new items within the framework of the diffusion model (Starns et al., 2018), because an item with a drift rate that deviates more strongly from zero tends, on average, to produce a faster (incorrect) response (lower RT; Ratcliff, 2014).^1^ At the same time, an item with a drift rate that deviates more strongly from zero is also more likely to result in the response indicated by the drift rate: a correct response for positive drift rates and an incorrect response for negative drift rates, when the upper and lower boundary represent correct and incorrect responses, respectively (Ratcliff et al., 2015). This also applies to the 2AFC task in which the memory signals of two presented items are compared (e.g., Kellen et al., 2021): The more strongly an item’s drift rate points toward the incorrect response boundary, the more likely it is to surpass the drift rate of the alternative item, thus more likely resulting in an incorrect decision.

The two-low threshold-model (2LTM; Starns, 2021; Starns et al., 2018) assumes that recognition responses arise from three discrete states. In a “detect old” state, the item is identified as previously studied leading to an “old” response. In a “detect new” state the item is identified as not studied, resulting in a “new” response. In the “uncertainty” state, no clear detection occurs and the response, “old” or “new,” is generated by guessing.^2^ Each path of the corresponding 2LTM-processing tree is entered with a conditional probability that depends on the identity of the item (studied vs. non-studied) or on the previous state. In the RT extended version of this model class, RT multinomial processing tree models (Klauer & Kellen, 2018), each path to a state is associated with its own processing time distribution. The sum of all processing times contributing to a specific decision process determines the observed RT (Klauer & Kellen, 2018). In the 2LTM, error responses result either from an incorrect detection (e.g., a response to a non-studied item in the “detect old” state) or from incorrect guesses. It can explain the error-speed effect by making two assumptions: first that an item entering a specific state in the first recognition task also enters the same state in the second recognition task; and second, that the processing times for the “incorrect detection” state are, on average, shorter than those for the “uncertainty” state (Heck & Erdfelder, 2016; Klauer & Kellen, 2018). If these two assumptions hold true, then for fast errors there is a higher proportion of responses from the “incorrect detection” state compared to slow errors resulting in a higher proportion of incorrect responses in the second recognition task.

Both of these models treat studied and non-studied items in a parallel manner. Thus, if the error-speed effect is expected to occur for studied items, it should also occur for non-studied items – although the effect size may differ (Starns et al., 2018). However, as mentioned above, this prediction was recently challenged by Akan et al. (2023), who observed the error-speed effect among misses but not among false alarms.

Although the absence of an error-speed effect among false alarms is somewhat surprising, it can, in principle, be accounted for by the above-outlined models. Considering the diffusion model, for example, the absence of an error-speed effect can be explained by a relatively large number of avoidable errors for non-studied items. In this scenario, most errors occur due to random fluctuations in the decision process and not because of systematically misleading memory evidence (Starns et al., 2018). A similar mechanism holds true for the 2LTM. If most false alarms arose from a state of “uncertainty” rather than from an “incorrect detection” state, there would be little to no error-speed effect.

Thus, observing no error-speed effect for false alarms indicates that false alarms arise solely based on random fluctuations in the decision process or based on guessing. This observation is particularly surprising in light of similarity effects in false recognition (e.g., Osth & Zhang, 2024; Shiffrin et al., 1995) and the predictions of many memory models (e.g., Gillund & Shiffrin, 1984; Hintzman, 1988; Osth & Dennis, 2015), which assume that recognition errors result from misleading memory evidence. Thus, it remains an open question whether the error-speed effect generally does not occur within false alarms, which would indicate that false alarms are generally a result of random fluctuation in the decision process or guessing, or whether it does occur when there is on average a higher amount of systematic misleading memory evidence.

Therefore, in the present study, we manipulated the similarity between studied and non-studied items. A higher similarity between studied and non-studied items should selectively increase the systematic misleading memory evidence for similar non-studied items (see, e.g., Meyer-Grant & Klauer, 2022; Shiffrin et al., 1995). According to the diffusion model, higher systematic misleading memory evidence is represented in a drift rate that deviates more strongly from zero (and points towards the incorrect decision boundary). Thus, including highly similar new items should increase the number of unavoidable errors and thereby lead to a more pronounced error-speed effect. By comparison, the 2LTM assumes that including new items with a stronger misleading memory evidence should increase the proportion of responses out of an “incorrect detection” state. If, typically, most false alarms arise from a state of “uncertainty,” increasing the proportion of errors from an “incorrect detection” state should lead to a more pronounced error-speed effect as it is strongest when false alarms arise in roughly equal proportions from an “uncertainty” state and an “incorrect detection” state. Thus, manipulating the similarity between studied and non-studied items allows us to discriminate between (a) whether false alarms are only the result of random fluctuations in the decision process or the result of guessing, and (b) whether false alarms can sometimes be the result of misleading memory evidence. In the former case, we would expect no error-speed effect for false alarms; in the latter case, we would expect an error-speed effect for false alarms when manipulating item similarity.

To directly manipulate similarity, we employed pictorial stimuli in the present study. Since previous studies have used only word stimuli, Experiment 1 first replicates the error-speed effect using picture stimuli using a design similar to Starns et al. (2018). In line with Akan et al. (2023), we observed the effect for misses but not for false alarms. In Experiment 2, we demonstrate, using a sequential-sampling test, that the error-speed effect occurs for false alarms when manipulating the similarity between studied and non-studied items.

## Experiment 1: Replication using pictorial stimuli

### Methods

The pre-registration, all materials to run the experiment, as well as the data and analysis scripts are available on the Open Science Framework (OSF) at: https://osf.io/jbqf9

#### Participants

We pre-registered to collect a minimum of 110 valid data sets. Given the absence of a firm expectation regarding whether the effect size for the error-speed effect with picture stimuli would be comparable to that observed with word stimuli, the sample size was adopted from the original study by Starns et al. (2018, Experiment 1).

Data were collected in a hybrid format: 19 participants were tested in the lab and 100 participants were tested online. Both versions used the same experimental code and sampled from the same participant pool, with participation being restricted to only one of the versions. The number of complete data sets was monitored at regular intervals. Data collection was terminated at the earliest point when the number of complete datasets equaled or exceeded the predefined sample size. If, after applying exclusion criteria, the number of valid data sets fell below the required threshold, data collection was continued.

This resulted in the final sample of 119 participants who completed the study. Two participants who did not conduct the task properly (guessing, almost exclusively using only one of the response keys) were excluded and five additional participants did not fulfill the inclusion criteria of a difference between hit rate and false alarm rate greater than .1. This resulted in 112 valid datasets. The final sample consisted of 84 female, 27 male, and one diverse participant, with age ranging from 18 to 41 years (*M* = 22.64, *SD* = 4.16).

Participants to both the online and lab study were recruited via the online platform Sona-Systems of the University of Freiburg. Eligible participants were aged between 18 and 45 years, fluent in German, and reported normal or corrected-to-normal vision. Participants who were tested online were instructed to complete the study in a quiet environment using a laptop or personal computer. Upon completion of the study, participants received partial course credit.

#### Material

As stimuli, we randomly selected pictures from a subset of stimuli used by Meyer-Grant and Klauer (2021).^3^ In total, 476 colour portrait pictures (depicting 238 females and 238 males) served as stimulus material, which had been generated by a generative adversarial network (Karras et al., 2019). Each picture had a resolution of 250 px × 300 px.

#### Procedure

During the experiment, participants encountered two types of cycles: one practice cycle and two experimental cycles. The practice cycle helped participants to become acquainted with the different tasks and the structure of the experiment. Practice and experimental cycles did not differ in their procedure. However, practice cycles consisted of fewer trials compared to experimental cycles. Only trials from experimental cycles were analysed.

**Fig. 1:**
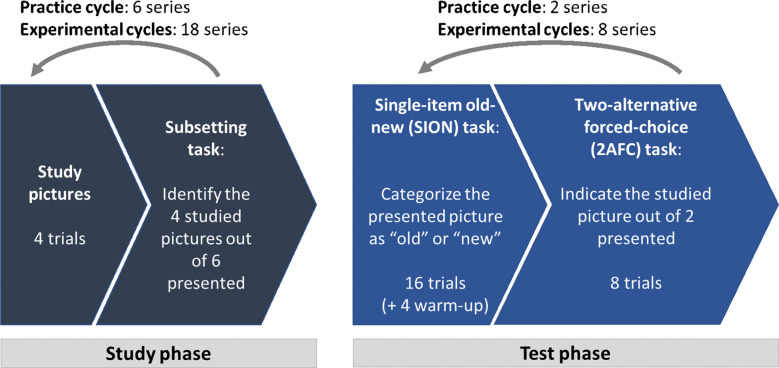
Study procedure for Experiment 1. The experimental cycle was replicated two times. The four warm-up trials in the SION task were only presented in the first series of each cycle

Each cycle combined a study phase and a subsequent test phase (see Fig. 1). In each study phase, participants were instructed to study portrait pictures presented sequentially at screen centre for 2,000 ms, separated by an inter-trial interval of 100 ms. After each block of four pictures (two images with male faces, two images with female faces), participants completed a subsetting task in which they were asked to select the four previously studied images from a set of six pictures (four studied and two non-studied; three male faces and three female faces). The purpose of this task was to encourage participants to remain attentive during the study phase. Following a blank screen (500 ms), the next block of study items was presented. In practice cycles, the study phase consisted of six blocks with four pictures each (24 pictures in total). In experimental cycles, participants encountered 18 blocks of four pictures (72 pictures in total). In both practice and experimental cycles, the first and the last block of four stimuli served to buffer primacy and recency effects, and were excluded from the test phase. This resulted in 16 valid stimuli for the test phase in the practice cycles and 64 in the experimental cycles.

Each test phase consisted of alternating SION blocks and 2AFC blocks. In the SION task, participants categorised the presented images as either being studied before (being old) or not (being new). Each stimulus remained on-screen until a response was recorded. To remind participants of the response mapping, the words “ALT” and “NEU” (German for “OLD” and “NEW”) were displayed below the stimulus, each paired with its corresponding response key (“M” for studied stimuli and “Y” for non-studied stimuli on a German QWERTZ keyboard). After an inter-trial interval of 100 ms, the next stimulus appeared.

In the 2AFC task, each trial presented a studied and a non-studied picture of the same sex side by side on the screen. Participants were required to indicate the position of the actually studied picture via keypress. The pictures were first shown individually for 1,000 ms, starting with the left one. Afterwards, both stimuli appeared together and remained on-screen until the participant responded. Participants used the “Y” key to indicate a studied picture on the left side and the “M” key to indicate a studied picture on the right side. As soon as both pictures appeared together on screen, labels were displayed that indicated the response mapping. After a 100-ms inter-trial interval, the next trial began.

Each SION block consisted of 16 trials including eight studied and eight non-studied pictures. However, the first SION block of each cycle included four additional warm-up trials, consisting of two old pictures drawn from the first four studied pictures and two new pictures. Pictures from these warm-up trials were not presented in the 2AFC task and were excluded from all data analyses. Overall, the sequence of old and new pictures in each block was randomised.

Each 2AFC block contained eight trials consisting of two pictures presented in the preceding SION block. In general, 2AFC trials could combine all possible types of previous SION responses (hit – false alarm; hit – correct rejection; miss – false alarm; miss – correct rejection).^4^ However, to test our hypothesis, it was essential to pair two pictures that had received the same SION response – one that had been previously answered correctly and one incorrectly. Therefore, these combinations (hit – false alarm; miss – correct rejection) were prioritised. The position of the studied picture (left/right) was counterbalanced within each block.

In total, participants completed two series of the SION task followed by the 2AFC task during the practice cycle. In the experimental cycle, eight such sequences of the SION and 2AFC task were conducted. To ensure that participants were prepared for responding, each SION block initially required a keypress of the “M” and “Y” key, which was followed by a 3-s countdown. Before starting a 2AFC block, a press of the space bar was required. Completion of the whole study took approximately 45 min.

#### Analyses

We considered the speed of recognition errors as the independent variable. Therefore, we categorised errors based on the individual median error speed, separately for studied and non-studied items. SION error responses that were faster than the respective median RT were categorised as fast errors, whereas error responses slower or equal to the respective median RT were considered slow errors.

To test for the presence of the error-speed effect, we conducted a repeated-measures ANOVA comparing arcsine-square root-transformed 2AFC accuracy (percentage correct) for fast and slow errors in the SION task, with error speed (fast vs. slow) and error type (miss vs. false alarm) as within-subject factors. Additionally, we conducted two separate paired *t*-tests that compared the arcsine-square root-transformed 2AFC accuracy for fast and slow errors to test for the occurrence of the error-speed effect separately for misses and false alarms.^5^

We complemented these analyses with corresponding Bayesian *t*-tests, following the prior specifications recommended by Klauer et al. (2025), which enable targeting of a prespecified effect size. For the present study, we chose an effect size of *d*_*z*_ = 0.3, which was the same effect size as we used in the sequential probability ratio *t*-test (SPRT) in Voormann et al. (2025). It represents the lower boundary of the 90% confidence interval when estimating a meta effect size over the results from our replication study on error-speed effects (Voormann et al., 2021).

### Results

#### Data preparation

We excluded trials with responses faster than 400 ms or slower than 8,000 ms. This led to the exclusion of 0.8% of trials. Descriptive statistics for the SION task are presented in Table 1. For analysis, we only considered critical 2AFC trials, the pairing of hit – false alarm (H – FA) and miss – correct rejection (M – CR). These pairings combined one item that had been responded to correctly with one item that had been responded to incorrectly in the SION task, such that both items had received the same previous response. The average number of critical trials was *M* = 50.8 (*SD* = 13.0) for misses and *M* = 26.4 (*SD* = 10.1) for false alarms.

**Table 1:** Mean response time, mean median response time, and proportion correct (PC) separately for response type in the single-item old/new (SION) task

SION response type	*M* (*SD*) in ms	*Md* (*SD*) in ms	PC (*SD*) in %
Studied correct (hit)	1,221 (283)	1,047 (212)	56.5 (11)
Studied error (miss)	1,256 (365)	1,078 (295)	
Non-studied correct (correct rejection)	1,168 (291)	1,001 (210)	76.0 (9)
Non-studied error (false alarm)	1,337 (423)	1,157 (354)	

#### Behavioural analysis

The repeated-measures ANOVA with the within-subjects factors error type (H – FA; M – CR) and error speed (fast vs. slow) using arcsine-square root-transformed proportion correct as dependent variable showed a significant error-speed effect. Items that elicited fast errors in the SION task were, on average, recognised less accurately in the subsequent 2AFC task (*M* =.68, *SD* =.14) compared to items that elicited slow errors (*M* =.72, *SD* =.12), *F*(1,111) = 7.65, *p* =.007, *η*_*p*_^*2*^ =.065. Additionally, there was a main effect of error type, *F*(1,111) = 46.21, *p* <.001, *η*_*p*_^*2*^ =.294, indicating less correct responses to 2AFC trials pairing a miss with a correct rejection (*M* =.66, *SD* =.11), than to trials pairing a hit with a false alarm (*M* =.74, *SD* =.14). The interaction between error type and error speed was not significant, *F*(1,111) = 3.09, *p* =.082, *η*_*p*_^*2*^ =.027. However, computing additional paired *t*-tests showed a significant error-speed effect for misses but not for false alarms (see Table 2). Fast misses were more often responded to incorrectly in a subsequent 2AFC task compared to slow misses. A Bayesian *t*-test confirmed these results. For misses, it was 2,391 times more likely that there was a difference in 2AFC performance with effect size *d*_*z*_ =.3 between fast and slow misses compared to no difference (BF_1,0_ = 2391). In contrast, for false alarms, it was 4.95 times more likely that no difference in 2AFC performance emerged between fast and slow false alarms compared to an effect of *d*_*z*_ =.3 (BF_0,1_ = 4.95).

**Table 2:** Mean proportion correct (PC; in %) and standard deviations for the critical trials in the 2AFC task as well as mean and standard deviations of response times (RTs; in ms) in the single-item old/new (SION) task separately for fast and slow responses in error and correct trials in the SION task with the results of the paired-samples t-tests

SION response type		Fast	Slow	*t*(111)	*p*	*d*_z_ [95% CI]
Studied error (miss)	PC	63.3 (11.2)	69.5 (10.1)	4.53	<.001	0.58 [0.31, 0.85]
	RT	860 (191)	1,644 (551)			
Non-studied error (false alarm)	PC	72.7 (14.8)	74.6 (13.6)	0.70	.484	0.08 [−0.15, 0.33]
	RT	919 (222)	1,736 (649)			
Studied correct (hit)	PC	76.8 (14.2)	70.7 (14.7)	3.08	.002	0.39 [0.13, 0.65]
	RT	865 (145)	1,549 (448)			
Non-studied correct (correct rejection)	PC	70.7 (10.7)	62.2 (9.4)	6.69	<.001	0.80 [0.53, 1.07]
	RT	823 (155)	1,506 (485)			

Our results replicate the results from Akan et al. (2023) using pictorial stimuli by showing an error-speed effect for misses but not for false alarms. However, the interaction between error-speed and error type reached significance only when using non-transformed error rates. Nevertheless, this still implies that erroneous decisions to old items are (partially) caused by misleading memory evidence while this is not the case for new items. As mentioned in the *Introduction*, the absence of an error-speed effect for false alarms may be due to systematically misleading memory evidence being rare in such errors, with most errors arising either from guessing (2LTM) or from random fluctuations in the decision process (diffusion model). To investigate whether false alarms can be caused by misleading memory evidence, we manipulated the similarity between studied and non-studied items in Experiment 2. As we were only interested to test whether the error-speed effect occurs for false alarms, we decided to use a sequential probability ratio *t*-test (SPRT) as it is typically more efficient for reaching a conclusion with pre-determined power than classical test regimes with a fixed sample size (Schnuerch & Erdfelder, 2020; see also Wald, 1945).

## Experiment 2: Manipulation of similarity

### Methods

We employed the same procedure as in Experiment 1 except for minor adjustments described below. The pre-registration, materials to run the experiment, as well as the data and analysis scripts are available on the OSF (https://osf.io/9g2uf).

#### Participants

We followed a sequential sampling plan using an SPRT which implements a *t*-test with an a priori specified power (1-β) and α-error given a specified effect size (*d*_*z*_). After every complete and valid data set the empirical likelihood ratio (LR), that is, the ratio of the likelihood of the observed data given the alternative hypothesis and the likelihood of the observed data given the null hypothesis, is compared to two decision criteria (for more details see Appendix in Voormann et al., 2025).

For the present study, we used the same effect size as in the Bayesian *t*-test of Experiment 1, *d*_*z*_ = 0.3. Additionally, we defined α =.05 and 1-β =.95. This resulted in the upper decision criterion *A* = 19 (evidence in favour of the alternative hypothesis – occurrence of the error-speed effect) and the lower decision criterion *B* = 0.053 (evidence for the null hypothesis – no error-speed effect in H – FA pairs). The inference criteria were checked at least once a day. As long as the empirical LR remained between these two criteria, sampling continued. Data collection stopped as soon as the LR crossed one of the two criteria. If a decision criterion was crossed while further data sets were sampled throughout the day, the inference decision was based on the data sets until the first crossing of one of the decision criteria.^6^

In total, data from 35 participants were collected. Two participants were excluded for not meeting the pre-registered inclusion criterion of a minimum difference of 0.1 between hit rate and false alarm rate, resulting in a final sample size of 33 participants. Among the participants, 27 identified as female, five as male, and one as diverse. Age ranged from 18 to 38 years (*M* = 23.7 years, *SD* = 4.8 years) and 30 participants (91%) reported being students. All participants were fluent in German and had normal or corrected-to-normal vision. Psychology students received partial course credit for their participation; all other participants received a monetary compensation of 8€.

#### Material

**Fig. 2:**
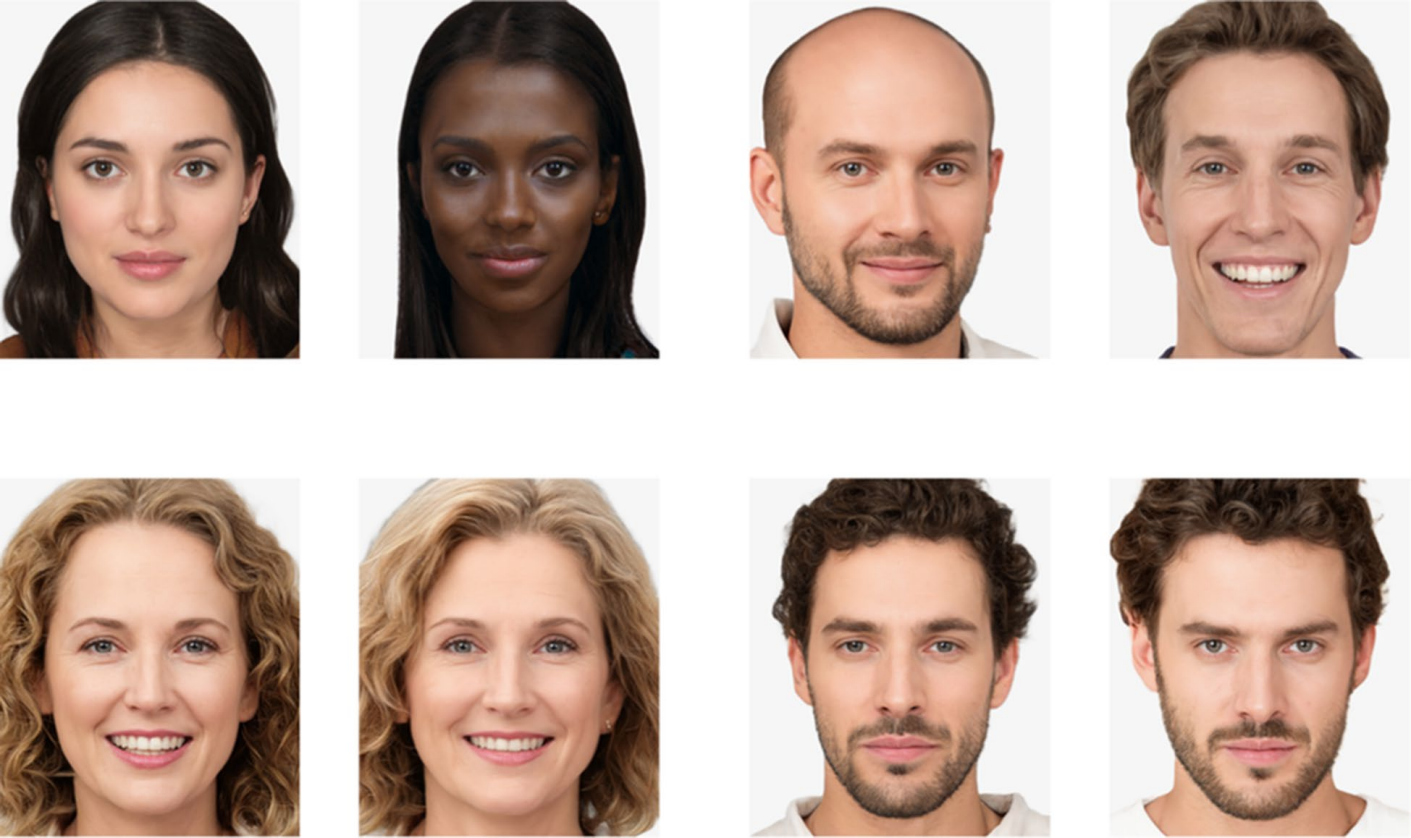
Illustrative items from the stimulus set with regular pairs in the top row and high-similarity pairs in the bottom row

We used the same picture stimuli as in Experiment 1. However, this time we included the similar picture pairs from Meyer-Grant and Klauer (2021), resulting in a final item pool of 632 colored portrait pictures depicting 316 females and 316 males. Within the complete set, 312 pictures formed 156 high-similarity pairs, each depicting two individuals of the same sex (78 female and 78 male pairs) that systematically resembled each other (for details, see Meyer-Grant & Klauer, 2021, Appendix B). The remaining 320 pictures, depicting 160 females and 160 males, had no systematic resemblance to one another. Figure 2 shows examples of two stimulus pairs with no systematic similarity between the depicted faces and two high-similarity stimulus pairs.

#### Procedure

**Fig. 3:**
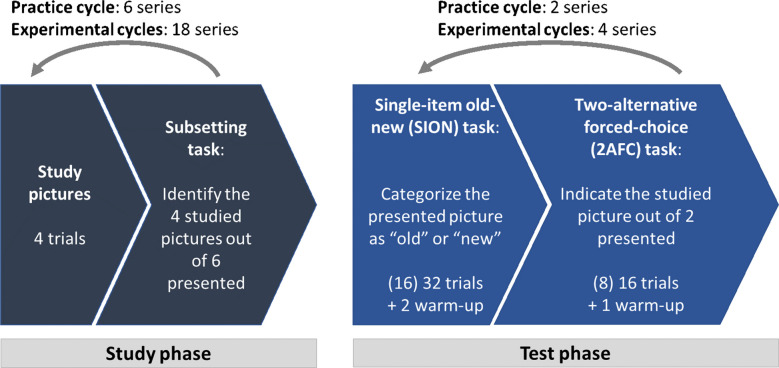
Study procedure for Experiment 2. The experimental cycle was replicated twice. The numbers in parentheses represent the trial number in the practice cycle

We kept the procedure similar to the previous experiment apart from small changes (see Fig. 3). In the study phase, half of the images were members of a high-similarity pair (with the remaining member not being presented during study), the other half was drawn from the item pool of regular pictures that exhibited no systematic resemblance to one another. The additional pictures for the subsetting task as well as for the first and the last block were drawn from an item pool consisting of the remaining regular pictures and one sibling randomly drawn from each remaining high-similarity pair. Half of the highly similar and regular pictures depicted male faces and the other half female faces. As we aimed to achieve as many critical pairs as possible in the test phase, we did not restrain sex on individual 2AFC trials and consequently also did not balance sex within each study block of four pictures but only across the whole study phase.

For the same reason, we also doubled the number of trials within each SION and 2AFC block in the experimental cycle and correspondingly halved the number of blocks per cycle. Therefore, participants worked through two SION blocks of 16 trials in the practice cycle (32 trials in total) and four blocks of 32 trials in the experimental cycles (128 trials per cycle). Each block started with two additional warm-up trials, an old picture taken from the first or last block during the study phase and a new picture. For each SION block, half of the trials presented an old stimulus, the other half a new stimulus. Furthermore, high-similarity and regular old and new pictures were equally frequent within each block. However, similar siblings, an old and a new picture belonging to the same high-similarity pair, never appeared in the same SION block, but always appeared two blocks apart from each other (e.g., the new picture appearing in block 1 and the matching high-similarity old picture in block 3). An inter-trial interval of 550 ms separated two consecutive SION trials.

For the 2AFC blocks, the two warm-up trials from each SION block were combined and presented at a random position within the 2AFC block. We constrained the composition of the other pairs such that each of the four possible combinations of high-similarity and regular old and new pictures were equally frequent. Again, critical pairs (combination of FA – H and M – CR) were prioritised, with the types of similarity pairings balanced as evenly as possible.

#### Analysis

We used the same procedure to categorise fast and slow errors as in Experiment 1, computing the median collapsed across highly similar and regular items. The reason for computing the median split across both highly similar and regular new items is that we expect (a) highly similar new items to produce relatively fast false alarms driven by strongly misleading memory evidence, and (b) regular new items to elicit slower false alarms based on weaker misleading evidence or incorrect guessing. As we were interested to test whether the error-speed effect occurs at all within new items, this contrast should best be captured by a median split across both kinds of items. Computing the median split separately for highly similar and regular lures would address a somewhat different question – namely, whether the effect also occurs *within* each item class. We also report results based on median splits separately for highly similar and regular lures in the *General discussion*.

The sequential sampling procedure was based on a *t*-test comparison of the mean arcsine-square root-transformed accuracy in FA – H pairs of the 2AFC task separately for fast and slow errors of the SION task. More precisely, to compute the LR we considered the *t*-value under the null and the alternative hypothesis. Our null hypothesis stated that there was no difference in mean arcsine-square root-transformed accuracy between fast and slow errors of the SION task. In contrast, our alternative hypothesis specified a difference with an expected effect size of *d*_*z*_ = 0.3.

In addition to the pre-registered sequential test, we computed a hierarchical logistic regression with the correctness of the 2AFC trials (correct: 1; incorrect: 0) as the dependent variable.^7^ To keep this analysis conceptually close to the sequential analysis, we included only FA – H pairs into the analysis. As predictors, we considered the error response speed (new items) and correct response speed (old items) in the SION task (z-standardised and log-transformed RT), the similarity of the new item (highly similar item: 1; regular item: 0), as well as their interactions. Analogous to Starns et al. (2018), we normalised RT distributions by taking the logarithm and standardised the log RTs separately for error and correct responses using the respective mean and standard deviation.

Furthermore, we included crossed random effects for participants, new pictures, and old pictures (Judd et al., 2012). We fitted the hierarchical logistic regression applying the “keep it maximal” principle proposed by Barr et al. (2013); we conducted a backwards selection for determining the random-effects structure.^8^ The *p*-values for fixed effects in the final model were determined via likelihood ratio tests.

### Results

#### Data preparation

Once again, we excluded trials with RTs in the SION task that were faster than 400 ms or slower than 8,000 ms. This led to an exclusion of 0.6% of total trials. Table 3 presents the descriptive statistics from the SION task for the complete sample. For analysis, we again considered only critical 2AFC trials. The average number of critical trials was *M* = 52.6 (*SD* = 15.0) for misses and *M* = 29.1 (*SD* = 9.6) for false alarms.

**Table 3:** Mean response time (RT) and proportion correct (PC) as well as standard deviations overall and separately for regular and high-similarity pictures per response type in the single-item old/new (SION) task

		Regular		High-similarity		Overall	
SION response type		RT in ms	PC in %	RT in ms	PC in %	RT in ms	PC in %
Studied correct (hit)	M (SD)	1,245 (271)	54.7 (14)	1,216 (296)	58.0 (13)	1,229 (278)	56.3 (13)
Studied error (miss)	M (SD)	1,251 (462)		1,308 (520)		1,282 (491)	
Non-studied correct (correct rejection)	M (SD)	1,170 (373)	85.5 (7)	1,276 (407)	64.1 (10)	1,215 (382)	74.8 (7)
Non-studied error (false alarm)	M (SD)	1,317 (492)		1,285 (352)		1,292 (384)	

*Note.* These descriptive statistics are computed on the complete sample and not only on the sample until a decision was reached by the sequential probability ratio *t*-test (SPRT)

#### Sequential probability ratio t-test

After collecting data from 28 participants, the likelihood ratio exceeded the upper boundary with LR = 20.5. Therefore, we accept the alternative hypothesis that the proportion of correct responses in 2AFC trials differs depending on whether the included new picture had previously elicited fast versus slow error response in the SION task. Consistent with the error-speed effect, new pictures that had led to fast errors in the SION task were associated with a lower proportion of correct responses in the subsequent 2AFC task than those that had led to slow errors (see Table 4).

**Table 4:** Mean response time (RT) in the SION task as well as mean proportion correct across both types (highly similar and regular) of new items (PC) and proportion of high-similarity pictures (P-HS) in the two-alternative forced-choice (2AFC) task separately for pictures responded to quickly or slowly in the single-item old/new (SION) task

	Fast			Slow		
SION response type	RT *M* (*SD*)	PC *M* (*SD*)	P-HS *M* (*SD*)	RT *M* (*SD*)	PC *M* (*SD*)	P-HS *M* (*SD*)
**Non-studied error** **(false alarm)**	**904 (194)**	**62.8 (13)**	**70.8 (12)**	**1679 (577)**	**71.6 (11)**	**71.7 (13)**
Studied error (miss)	899 (297)	62.0 (11)	45.2 (10)	1,661 (694)	65.8 (11)	51.7 (13)
Non-studied correct (correct rejection)	847 (188)	68.4 (13)	42.6 (7)	1,608 (573)	59.6 (10)	53.4 (9)
Studied correct (hit)	884 (152)	71.6 (13)	53.6 (12)	1,574 (413)	62.6 (17)	49.2 (12)

*Note.* The row containing the relevant results from false alarms are indicated in bold font. These descriptive statistics are computed on the complete sample. However, they are similar in the sub-sample on which the inference decision is based in the sequential probability ratio *t*-test (SPRT)

#### Hierarchical logistic regression

Table 5 shows the result for the final model. The fixed effects of response speed in SION for both new and old items were statistically significant. As the analysis included only H – FA pairs, the error response speed indicates the error-speed effect for new items. Faster error responses (lower RTs) led to a decreased probability of a correct response in the 2AFC task. For correct items the effect was reversed with faster correct responses, leading to an increased probability of a correct response, attesting to the correct-speed effect. Furthermore, there was a statistically significant effect of similarity. H – FA pairs including a regular new item had a higher probability of a correct response compared to H – FA pairs including a highly similar new item. Most importantly the interaction between similarity and error response speed was not significant, indicating that there was no significant difference in the error-speed effect for regular and highly similar new items.

**Table 5:** Regression weights (estimates and standard error), the likelihood ratio test statistic, and corresponding *p*-values for the fixed effects of the hierarchic logistic regression predicting the correctness of two-alternative forced-choice (2AFC) trials

	Estimate (SE)	${\chi }^{2}$(1)	*p*
Similarity	−0.59 (0.18)	11.02***	<.001
ErrorLogRT	0.45 (0.19)	6.21*	.013
CorrectLogRT	−0.32 (0.15)	4.68*	.030
Similarity $\times$ ErrorLogRT	−0.20 (0.21)	0.94	.331
Similarity $\times$ CorrectLogRT	0.05 (0.17)	0.08	.771
ErrorLogRT $\times$ CorrectLogRT	−0.12 (0.16)	0.54	.598
Similarity $\times$ ErrorLogRT $\times$ CorrectLogRT	−0.14 (0.19)	0.53	.465

*Note.* *** *p* <.001, * *p* <.05

## General discussion

In Experiment 1, we replicated the findings from Akan et al. (2023) using pictorial stimuli, demonstrating that the error-speed effect occurs for misses but not for false alarms. For misses, responses in the 2AFC task were, on average, less accurate when the trial included a stimulus that had previously elicited a fast erroneous response in the SION task compared to when it included a stimulus associated with a slow response. However, for false alarms our results suggested no such difference. Since the error-speed effect is generally interpreted as evidence for recognition errors being (partially) based on misleading memory evidence, its absence suggests that errors arise solely from either random fluctuations in the decision process (diffusion model) or guessing (2LTM). Thus, the findings of Experiment 1 and the results from Akan et al. (2023) seem to suggest different sources for errors depending on the item’s identity (old vs. new).

To investigate whether false alarms can, in principle, result from misleading memory evidence, we manipulated the similarity between some old and new items in Experiment 2. By including new items that had a similar old-item counterpart, we found evidence supporting the occurrence of an error-speed effect for false alarms. This suggests that, under the right conditions, even false alarms are (at least partially) driven by misleading memory evidence and are not solely the result of random fluctuation or guesses. Importantly, in the hierarchical logistic regression there is no statistical difference in the error speed effect for highly similar and regular new items.

This finding is in line with the diffusion model and the 2LTM, which both tend to predict the error-speed effect for misses *and* false alarms.^9^ As mentioned in the *Introduction*, both of these models suggest that including highly similar new items should increase the error-speed effect because they increase the variance in systematic misleading memory evidence across all new items. The diffusion model predicts this pattern through a higher variability in the drift rate when highly similar and regular new items are included, compared to when only regular new items are present due to a higher variance in misleading memory evidence. This, in turn, results in a higher variability in the probability for a correct response, which in combination with the median split into fast and slow errors results in a more pronounced error-speed effect. By comparison, the 2LTM predicts a more pronounced error-speed effect when false alarms arise in roughly equal proportions from an “uncertainty” state and an “incorrect detection” state. Assuming that most responses to regular new items come out of a state of uncertainty, including highly similar new items should increase the proportion of incorrect detections and should also increase the error-speed effect for false alarms.

Thus, the diffusion model and the 2LTM are both able to predict why the error-speed effect for false alarms arises when manipulating the similarity between studied and non-studied items and why it may not arise in a case with only regular new items. Nevertheless, the question remains whether only false alarms in highly similar new items are partially elicited by misleading memory evidence leading to the error-speed effect or whether false alarms in regular new items, too, can occur based on misleading memory evidence. When looking at the effect sizes in the complete sample for the error-speed effect with the combined median split separately for highly similar (*d*_*z*_ = 0.44, 95% confidence interval for the standardised effect size (CI) [−0.10, 0.99]) and regular new items (*d*_*z*_ = 0.39, 95% CI [−0.10, 0.87]), we descriptively observe little difference between the two types of new items, but only a difference of both effect sizes compared to the overall effect size of *d*_*z*_ = 0.71, 95% CI [0.17, 1.24], reflecting the smaller numbers of trials and the greater impact of measurement error in the separate analyses than in the overall analysis. Please note, however, that due to the sequential sampling plan effect size estimates can be biased. Using separate median splits for highly similar and regular new items, we observe even a descriptively slightly larger effect size for regular new items *d*_*z*_ = 0.50, 95% CI [0.01,1.00], compared to highly similar new items *d*_*z*_ = 0.43; 95% CI [−0.13, 0.98]. The existence of the error-speed effect within regular new items indicates that false alarms in regular new items are also partially elicited by misleading memory evidence and not only false alarms of highly similar new items. However, the core objective of this study was to determine whether false alarms can be elicited by misleading memory evidence. This question can be most effectively addressed by employing a median split across both categories of new items (i.e., regular and highly similar ones). Computing the median split separately for highly similar and regular lures addresses a different question: whether the effect also occurs *within* each new-item category. It is not surprising that the effect sizes within each item category are smaller compared to the effect sizes across both item categories for two reasons: First, we assumed that, on average, highly similar new items are associated with higher levels of misleading memory evidence than regular ones. Thus, highly similar new items are expected to elicit relatively fast false alarms, whereas regular new items should typically produce slower false alarms. Computing separate median splits for the two types of new items artificially removes this desired contrast between conditions.^10^ Second, there are fewer trials per condition which should increase the noise in the estimation of the error-speed effect.

Interestingly, a similar picture arises for the proportions of highly similar new items within fast (70.8 %) and slow (71.7 %) false alarms in Table 4, which are roughly equivalent across fast and slow false alarms. This is quite surprising as both models, the diffusion model and the 2LTM, would suggest that items associated with higher misleading memory evidence preferentially end up as fast errors rather than as slow errors. However, a descriptive assessment of the mean RTs for false alarms in Table 3 hints at a difference in the correct direction with a mean RT of 1,285 ms for highly similar pictures and a mean RT of 1,317 ms for regular pictures. Nevertheless, the similar proportions suggest that false alarms arise partially because of misleading evidence both in highly similar new items and in regular new items. This interpretation is further supported by the observation that the error-speed effect in lures is not significantly moderated by similarity in the hierarchical logistic regression, indicating that the error-speed effect does not differ significantly between highly similar and regular new items.

The absence of a difference in the proportions might be a byproduct of the small numbers of critical trials including a false alarm (*M* = 29.1) and the limited test power for detecting a difference implied thereby. The relatively small number of critical 2AFC trials including false alarms compared to those including misses (*M* = 52.6 in Experiment 2) may also contribute to the observed asymmetry: the error-speed effect being reliably observed for misses but not for false alarms. In passing, our manipulation also addresses this second point by slightly increasing the number of critical trials that included a false alarm from *M* = 26.4 to *M* = 29.1.

Based on the present data, it is not possible to determine with certainty whether the occurrence of the error-speed effect for false alarms is due to more misleading memory evidence, a higher number of critical trials, or a combination of the two factors. Ultimately, however, this distinction does not affect the conclusion that all types of recognition errors are at least partly attributable to misleading memory evidence, even false alarms.

## Data Availability

We preregistered the analyses of Experiment 1. The pre-registration, all materials to run the experiments, the data, and data analysis script can be found on the OSF (https://osf.io/jbqf9). Additionally, we pre-registered the methods and analyses of Experiment 2. The pre-registration, all materials to run the experiment, the data, and data analysis script can be found on the OSF (https://osf.io/9g2uf).

## Footnotes

1 Please note that when we use the term “fast error” we are not referring to particularly fast errors that are in the framework of the diffusion model accounted for by the combination of a varying starting point of the accumulation process across trials and random fluctuations of noise in the sampling process (Ratcliff & Tuerlinckx, 2002). Instead, we distinguish between fast and slow errors using a median split.

2 Please note that the 2LTM is very similar to the well-known two-high threshold-model (2HTM; Snodgrass & Corwin, 1988). However, unlike the 2HTM it allows for incorrect detections, meaning that it considers a “detect old” state for new items and a “detect new” state for old items, whereas in the 2HTM all errors are the result of incorrect guesses.

3 Note that we only selected one of the pictures of each high-similarity pair used by Meyer-Grant and Klauer (2021), ensuring that no systematically similar pictures were included in Experiment 1.

4 A hit is a correct response to an old item whereas a correct rejection is a correct response to a new item. As noted earlier, a miss is an incorrect response to an old item whereas a false alarm is an incorrect response to a new item.

5 Please note, we did not pre-register the two *t*-tests. However, Akan et al. (2023) demonstrated that the interaction between error speed and error type might not be significant although there was an effect in misses but not in false alarms. Thus, we decided that it was necessary to replicate these findings for picture stimuli. Additionally, we applied arcsine-square root-transformed proportion correct of the 2AFC task, which was a deviation from our pre-registered analysis plan. This transformation changed some values numerically, but did not change the main statistical conclusions except that the interaction between error speed and error type was significant with *F*(1,111) = 4.34, *p* =.040, without the transformation.

6 To ensure feasibility, we defined a maximum sample size of *N* = 122. If the maximum had been reached without an inference decision, we would have reported the observed LR as a descriptive measure of the statistical evidence provided by the data but refrained from conducting any additional inference tests. However, one of the criteria was already crossed after 28 valid data sets.

7 This analysis was conducted in response to a comment by a reviewer and was not pre-registered.

8 For feasibility reasons, we first conducted three separate backwards model selection procedures including only one of the three random-effect factors (i.e., participants, new picture, or old picture). Each of those three selection procedures started with the respective maximal random-effect structure, that is, all random-effects but not their correlations were initially included. If a model failed to converge or showed a singular fit, we systematically reduced the random-effects structure based on the least incremental explanatory value (in terms of variance accounted for). However, exclusion did not violate the principle of marginality and the correlations between random effects were always excluded first. The maximal random-effects structures for each of the three random-effects factors that converged and led to a nonsingular fit were then combined and the resulting model served as a starting point for a final model selection procedure containing all three random-effect factors. This was accomplished by yet another backwards selection adhering to the same principles as the previous ones. The final model included in its random effects structure only a random intercept for the old pictures and a random intercept for the new pictures.

9 The diffusion model even predicts an interaction between error speed and error type, as the error-speed effect is expected to be smaller for false alarms compared to misses (Starns et al., 2018).

10 Consider the most extreme case within the 2LTM, in which all errors for highly similar new items arise from an incorrect detection state, whereas all errors for regular new items result from incorrect guessing. Even if a genuine and robust error-speed effect exists, conducting separate median splits for highly similar and regular new items would obscure this effect, ultimately leading to the erroneous conclusion that no error-speed effect is present.

## Code availability

Data analysis script of Experiment 1 can be found on the OSF (https://osf.io/jbqf9). Data analysis script of Experiment 2 can be found on the OSF (https://osf.io/9g2uf).
